# Evaluation of the Novel Bacteriophage Chage1 and Its Endolysin LysCG1 as Biocontrol Agents against *Cronobacter sakazakii* in Foods

**DOI:** 10.4014/jmb.2601.01065

**Published:** 2026-03-26

**Authors:** Jin Seo, Minsuk Kong

**Affiliations:** Department of Food Science and Biotechnology, Seoul National University of Science and Technology, Seoul 01811, Republic of Korea

**Keywords:** *Cronobacter sakazakii*, Bacteriophage, Powdered infant formula (PIF), Biocontrol, Endolysin

## Abstract

*Cronobacter sakazakii* is a foodborne pathogen transmitted through powdered infant formula (PIF) and consequently leads to illness in infants. Owing to the pathogen’s occasional antibiotic resistance, bacteriophages offer a promising strategy for controlling this pathogen. Therefore, this study characterized a novel virulent phage (Chage1) that specifically infects *C. sakazakii* cells. Chage1 exhibited stability across a temperature range of 4–50°C and pH conditions of 4–11. Moreover, Chage1 inhibited bacterial growth for up to 24 h when inoculated into *C. sakazakii* at a multiplicity of infection (MOI) of 1. Chage1 (MOI of 10^5^) also showed antibacterial activity against two *C. sakazakii* strains in PIF and milk samples for 8 h at 37°C. Whole genome sequencing of Chage1 revealed a 52,653 bp DNA genome with 72 predicted open reading frames (ORF) containing an endolysin gene (ORF 60). The endolysin LysCG1 was fused with a maltose-binding protein for solubility and its lytic activity against various bacterial strains was evaluated. LysCG1 exhibited a broader lysis spectrum than that of Chage1 by inhibiting the growth of diverse ethylenediaminetetraacetic acid-pretreated Gram-negative bacterial cells. Overall, this study provides a basis for the potential application of Chage1 and its endolysin for the control of *C. sakazakii* in food production.

## Introduction

*Cronobacter sakazakii* is a facultative anaerobic, Gram-negative, foodborne pathogen that has been detected in diverse food products [1]. This bacterium contaminates infant formula, flour, and dairy products and exhibits substantial resistance to environmental stressors, such as acidity, heat, and dryness [2-4]. *C. sakazakii* survives in powdered infant formula (PIF) for up to 2.5 years [5]. Moreover, its ingestion leads to life-threatening conditions, such as necrotizing enterocolitis, sepsis, and meningitis in immunocompromised infants [6, 7], with mortality rates reaching 50–80% [8, 9]. For instance, a CDC report described two infants in the United States with *C. sakazakii* infections in 2021-2022, one associated with PIF and the other with feeding equipment; these infections presented as invasive disease, with one infant surviving after treatment and the other resulting in death, highlighting the serious clinical consequences of infant food contamination [10]. Consequently, the development of safe and effective biological control strategies for managing *C. sakazakii* infections is a critical challenge.

Bacteriophages are viruses that lyse bacterial cells in specific hosts. Owing to their high selectivity, phages only infect the target bacteria or closely related strains within the same species. Phages are abundant, safe, and possess excellent antimicrobial properties, rendering them promising alternatives for biological control in the food industry [11]. Phage-derived endolysins also represent another promising antimicrobial agent. This enzyme hydrolyzes the peptidoglycan layer of the bacterial cell wall, leading to cell lysis [12]. Although an endolysin exhibits specificity toward its bacterial host, it often demonstrates a broader lytic spectrum than that of the phage itself [13]. In addition, the risk of developing resistance against endolysin is low compared to that against bacteriophages, thereby reinforcing its potential as a promising antimicrobial candidate [14, 15]. Considering that *C. sakazakii* occasionally shows resistance to antibiotics, the use of bacteriophages or phage-derived proteins, such as endolysins, may offer a highly effective strategy for controlling this pathogen.

Although several *C. sakazakii*-infecting phages have been isolated and characterized, those capable of infecting the reference strain *C. sakazakii* BAA-894 remain relatively rare. This suggests that this strain is relatively resistant to many naturally occurring *C. sakazakii* phages [16-18]. In this study, a virulent phage (Chage1) that infects *C. sakazakii* BAA-894 was isolated from a wastewater treatment plant and its morphological and biological characteristics were investigated. To assess the phage’s applicability, the antimicrobial activity of Chage1 against *C. sakazakii* was evaluated in milk and PIF. Simultaneously, Chage1 genomic information and functional genes were analyzed using whole-genome sequencing to exclude potential risks. Additionally, the endolysin LysCG1 was isolated and purified from Chage1 and its antimicrobial spectrum was characterized. Consequently, the study evaluated the potential of Chage1 and LysCG1 as novel biological control agents against *C. sakazakii* infections.

## Materials and Methods

### Bacterial Strains and Growth Conditions

All bacterial strains used in this study are listed in Table 1. The *C. sakazakii* isolates were obtained from Seoul National University [19]. *E. coli* BL21 (DE3), transformed with a recombinant plasmid, was cultured in Luria-Bertani (LB) broth supplemented with 50 μg/ml kanamycin at 37°C. *Listeria monocytogenes* and *Staphylococcus aureus* were grown in Tryptic Soybean Broth (TSB) at 37°C with shaking at 250 rpm. *Bifidobacterium adolescentis* was cultured statically in Brain Heart Infusion (BHI) broth within an anaerobic chamber (A25 Anaerobic workstation IAP, Don Whitley Scientific Limited, UK) at 37°C under an atmosphere of 10% H_2_, 10% CO_2_ and 80% N_2_. *Lactobacillus paracasei* was grown in Lactobacilli MRS broth with agitation, while *Lactobacillus rhamnosus* was grown in MRS broth under anaerobic conditions as described above. All other bacterial strains were grown under agitation at 37°C in LB broth overnight. Agar media were prepared by supplementing the respective broth medium with 1.5% agar. Bacterial stocks were stored at -80°C with 15% glycerol.

### Phage Isolation and Purification

Phage Chage1 was isolated and purified using a modified procedure based on a previous study [20]. A 10 ml sewage sample (Guri sewage treatment, Republic of Korea) was mixed with 10 ml of 2× LB broth supplemented with 5 mM MgCl_2_ and CaCl_2_. An overnight culture of *C. sakazakii* BAA-894 was inoculated into the mixture and incubated overnight at 37°C with agitation. After centrifugation (10,000 g) for 20 min, the supernatant (phage-enriched solution) was filtered through a 0.45 μm membrane filter (Sartorius AG, Germany) and spotted onto a double-layer LB agar plate containing *C. sakazakii* BAA-894. The overlay assay was repeated five times to isolate a single phage. The isolated phage was then propagated by incubating with the host strain at 37°C for 3 h with shaking at 250 rpm. Phage particles were precipitated with 10% polyethylene glycol 6000 and 1 M NaCl at 4°C overnight. Following centrifugation, the pellet was resuspended in sodium chloride-magnesium sulfate buffer (SM buffer; 50 mM Tris-HCl, 8 mM magnesium sulfate, 100 mM sodium chloride, pH 7.5) and further purified by cesium chloride (CsCl) density gradient centrifugation (78,500 g, 2 h). The purified phage was dialyzed using SnakeSkin dialysis tubing (molecular weight cut off 3.5 kDa, Thermo Fisher Scientific, USA) for 2 h.

### Transmission Electron Microscopy (TEM)

The morphological analysis was conducted as previous studies [20]. A 10 μl drop of purified phage Chage1 was placed on a copper mesh grid (formvar/carbon coated TEM grid, 200 mesh) and incubated at room temperature for 1 min. The excess liquid was removed with filter paper, and the grid was stained with 10 μl of 2% uranyl acetate (pH 4.0) for 1 min. Excess stain was similarly removed. Phage morphology was observed using energy-filtering transmission electron microscopy (Libra 120, Carl Zeiss, Germany) operated at 80 kV. The head and tail dimensions were measured from TEM images using ImageJ software.

### Host Range Analysis

The host range of Chage1 was analyzed as described previously, with some modifications [16]. Various bacterial strains listed in Table 1 were used in this study. Overnight bacterial culture 100 μl was added to 0.4% distilled water (D.W.) soft agar (5 ml) with 5 mM MgCl_2_ and CaCl_2_, and then mixture was poured into LB agar plate. 10-fold serial dilutions of the phage suspension were spotted onto double-layered agar plate, and the plates were incubated at 37°C to observe whether a single plaque appears.

### Adsorption Assay

The adsorption assay was conducted as described previously, with some modifications [16]. Phage Chage1 was mixed with early exponential phase *C. sakazakii* BAA-894 at a multiplicity of infection (MOI) of 0.01 and incubated at 37°C. At 2, 5, 10, 15, and 20 min, aliquots were centrifuged, and the number of unbound phages in the supernatant was quantified by double-layered agar plate method. The phage adsorption percentage is based on the calculation formular as follows:


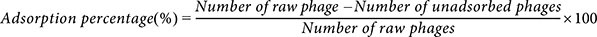


### One-Step Growth Curve

The one-step growth curve was constructed with a modified method [2]. *C. sakazakii* BAA-894 in the early exponential phase was mixed with phage Chage1 at an MOI of 0.01, followed by incubation in a 37°C heat block for 2 min to allow phage adsorption. Unadsorbed phages were removed by centrifugation (13,000 g, 1 min), and the pellet was washed twice with LB broth. The pellet was then resuspended in 10 ml of LB broth and incubated at 37°C. Samples (300 μl) were collected every 10 min for 120 min, with or without 1% chloroform, immediately diluted, and plated to determine phage titers. Results were reported as average phage titers, and the burst size was computed as the ratio of the amount of released phage particles to the initial amount of infected bacterial cells as described previously [21].

### pH and Thermal Stability

To determine the stability of Chage1 under various temperature and pH conditions, phage suspensions (10^8^ PFU/ml) were incubated under different conditions. Thermal stability was assessed by incubating the phage at temperatures ranging from 4 to 70°C for 3 h. For pH stability, 100 μl of phage lysate was mixed with 900 μl of Britton-Robinson universal pH buffer (40 mM boric acid, 40 mM phosphoric acid, 40 mM acetic acid, pH 2-12) and incubated for 1 h. Phage viability was determined by quantifying the remaining active phages using the double-layered agar plate method.

### Bacterial Challenge Test

A 950 μl aliquot of *C. sakazakii* ATCC 29544 bacterial suspension (10^8^ CFU/ml) was added to a sterile 24-well plate. For the experimental groups, 50 μl of phage Chage1 solution was added at MOIs of 0.01, 0.1 and 1. In the control group, 50 μl of SM buffer was added instead. The OD_600_ was measured every 15 min for 24 h at 37°C using a multi-mode microplate reader (SpectraMax i3x, Molecular Devices, USA).

### Food Application

The antibacterial effect of Chage1 in food sample was evaluated as previously described, with minor modifications [2, 22]. Sterilized commercial milk and powdered infant formula (PIF) were purchased online. The milk and PIF were plated on LB agar in advance to confirm that no bacteria were detected. *C. sakazakii* BAA-894 and ATCC 29544 were cultured and centrifuged at 13000 g for 1 min. The supernatant was removed, and the pellet was resuspended in PBS buffer. Bacterial suspension (1 ml) diluted to 10^5^ CFU/ml, was added to 9 ml of milk, and the mixture was then treated with Chage1 phage at an MOI of 10^5^. Samples were incubated at 37°C with shaking for 8 h, and 300 μl of the mixture was serially diluted with PBS and plated on the LB agar for colony counting.

### Phage DNA Extraction and Purification

Genomic DNA of Chage1 was extracted using the phenol-chloroform method, as described previously [23]. Briefly, purified phage lysates were treated with DNase I and RNase A to remove host nucleic acids, followed by proteinase K and sodium dodecyl sulfate (SDS) treatment for capsid digestion. After phenol-chloroform extraction, DNA was recovered by ethanol precipitation and resuspended in distilled water.

### Phage Genome Sequencing and Genomic Analysis

The genomic DNA of Chage1 was sequenced using the Illumina NextSeq V3 platform and assembled with SPAdes (v3.15.2), and annotated using Rapid Annotation on the Subsystem Technology server [24]. Additional annotations were performed with BLASTp (https://blast.ncbi.nlm.nih.gov/Blast.cgi) and InterProScan databases (https://www.ebi.ac.uk/interpro/search/sequence/). Virulence factors and antibiotic resistance genes were identified using the VFDB (https://www.mgc.ac.cn/VFs/) [25] and the CARD database (https://card.mcmaster.ca/)[26], respectively. Whole-genome ANI of each bacteriophage was calculated using an ANI calculator (https://www.ezbiocloud.net/tools/ani). Phylogenetic analysis was conducted using MEGA 12 [27] to determine evolutionary relationships. Comparative genomic analysis was performed using Clinker (v0.0.23) [28]. Amino acid sequences of tail fiber proteins were aligned using Clustal X 2.0, and the alignments were visualized with GeneDoc to compare sequence conservation and divergence among Chage1 and related phages [29]. The complete genome sequence of Chage1 has been deposited in the NCBI Genbank database under the accession number PQ498404.

### Construction of Plasmid and Overexpression

The maltose binding protein (MBP) gene was PCR amplified using 10His-pET28a::MBP [30] as a template and the primers fNcoI_MBP (5’-GCGCCATGGGCATGAAAATCGAAGAAGGTAAACTGGTAATCT-3’) and rNcoI_MBP (5’-GCGCCATGGGCCCGAGGTTGTTGTTATTGTTA-TTGTTGT-3’). The PCR product was digested with NcoI enzyme and ligated into the pET28a expression vector. The endolysin gene (ORF60) from phage Chage1 was amplified using primers fBamHI_LysCG1 (5’-GCGGGATCCATGAGTCTCAAATCAACAGCGACTAAAGC-3’) and rHindIII_LysCG1 (5’-GCGAAGCTTTTATTGCGATTGCTGCACTGGTTT-3’). The resulting PCR product was digested with BamHI and HindIII, and cloned into pET28a::MBP vector.

### Turbidity Reduction Assay

Bacterial cells were grown to the exponential phase (approximately OD_600_ = 1.0) and treated 100 mM ethylenediaminetetraacetic acid (EDTA) for 10 min to permeabilize the outer membrane. The cells were then washed twice using reaction buffer (20 mM Tris-Cl, pH 8.0) by centrifugation at 13000 g for 1 min, and the resulting pellet was resuspended in reaction buffer. Purified endolysin (LysCG1) was added to a final concentration of 1.0 μM, and the OD_600_ values were monitored over time at 25°C using a SpectraMax i3x plate reader (Molecular Devices, USA). Relative lytic activity was calculated at the lowest OD_600_ value of the endolysin-treated group using the following equation:


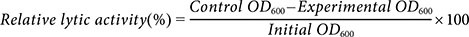


### Statistical Analysis

All experiments were conducted in triplicate and the data were presented using mean values and standard deviations. Statistical significance was evaluated using Student’s t-test and one-way ANOVA, and differences were considered significant at *p* < 0.05. All statistical analyses were performed GraphPad Prism 5.0 software (GraphPad, USA)

## Results

### Isolation of Phage Chage1 and Its Host Range

**Fig. 1:**
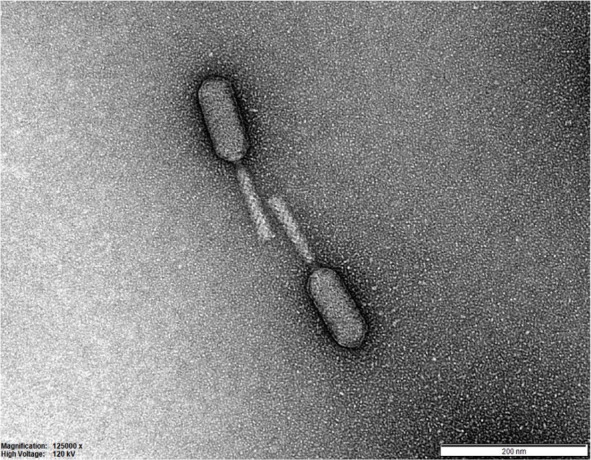
Morphology of Chage1. TEM showed that Chage1 has a head size of 104.0 ± 6.6 nm [*n*= 25], tail size of 94.6 ± 8.1 nm [*n* = 25] with contractile characteristics.

Chage1 that infects *C. sakazakii* BAA-894 was isolated and purified from a sample collected from a sewage treatment plant in Guri City, Korea. TEM analysis (Fig. 1) revealed that Chage1 (*n* = 25) has a prolate head (104.0 ± 6.6 nm) and a contractile tail (94.6 ± 8.1 nm), which are typical morphological features of the class *Caudovirales*. To evaluate the potential of Chage1 as an antimicrobial agent, its host range was determined using a double-layered agar method. Chage1 infected 4 out of 11 tested *C. sakazakii* strains by forming small and transparent plaques, which are characteristic of virulent phages. In contrast, other *Cronobacter* species were resistant to Chage1 phage but exhibited the inhibition zones instead of plaque formation. Notably, the host range of Chage1 does not affect beneficial gut bacteria, such as *Bifidobacterium* and *Lactobacillus*, thus ensuring its safety for use in food applications without affecting other probiotic bacteria.

### One Step Growth Curve and Challenge Assay (Growth Characteristics)

**Fig. 2:**
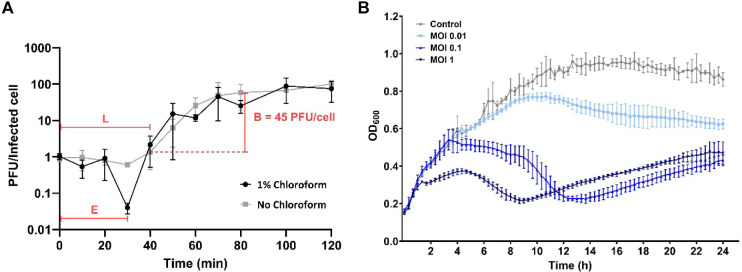
Growth characteristics of Chage1. (**A**) One-step growth curve of Chage1. Chage1 was grown in an exponential phase culture of *C. sakazakii* BAA-894. The phage growth parameters are indicated as follows: E, Eclipse period; L, Latent period; B, Burst size. (**B**) The growth of *C. sakazakii* ATCC 29544 was periodically monitored for 24 h after treatment with Chage1 at the indicated MOIs.

Chage1 exhibited an approximately 95% adsorption percentage within 2 min (Fig. S1). The one-step growth curve indicated that the eclipse period was 30 min, the latent period was 40 min, and the burst size was 45 PFU per infected cell upon infection of *C. sakazakii* BAA-894 cells (Fig. 2A). The host lytic activity of Chage1 was determined using a bacterial challenge assay, and the phage effectively inhibited the growth of *C. sakazakii* for 9 h at an MOI of 1 in LB broth. However, after 9 h, Chage1-resistant bacteria emerged and began to grow slowly (Fig. 2B). Furthermore, at lower phage concentrations, the growth of the host strain was weakly inhibited (Fig. 2B).

### Stability of Chage1

**Fig. 3:**
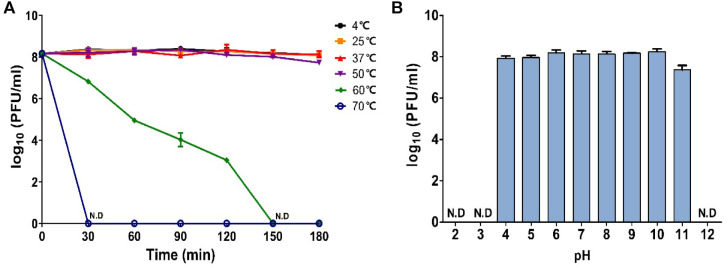
Effects of temperature and pH on the stability of phage Chage1. (**A**) The thermal tolerance of Chage1 was investigated across a temperature range of 4°C to 70°C. (**B**) pH stability was determined within the pH range of 2 to 12.

The thermostability of Chage1 is shown in Fig. 3A. The titer of Chage1 remained relatively stable at approximately 8 log PFU/ml across the temperature range of 4–50°C. When cultured at 60°C, Chage1 titer declined to 5 log PFU/ml after 60 min and became undetectable after 150 min, while complete inactivation occurred at 70°C. Chage1 maintained a relatively stable high titer of 8 log PFU/ml in the pH range of 4–10 (Fig. 3B). Beyond this range, the titer decreased: at pH 11, Chage1 exhibited an activity of 7 log PFU/ml, whereas it was completely inactivated at pH 3 or above 12.

### Lytic Activity of Chage1 in Food Samples

**Fig. 4:**
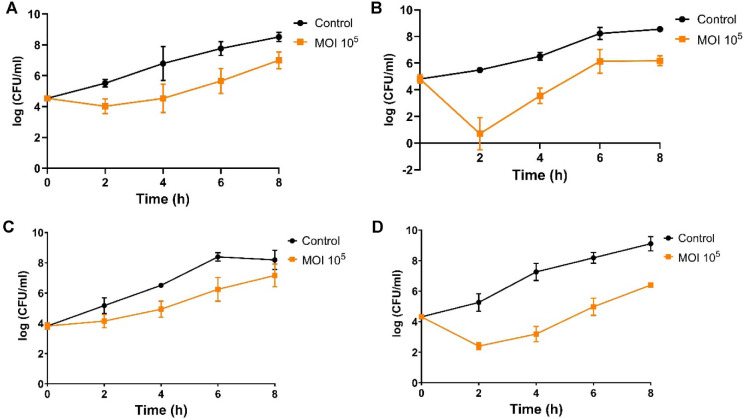
Antimicrobial efficacy of Chage1 in food matrices. Chage1 demonstrated antibacterial activity on *C. sakazakii* BAA-894 (**A**) and ATCC 29544 (**B**) in sterilized skim milk for 8 h at 37°C. Furthermore, Chage1 exhibited antibacterial properties against both *C. sakazakii* BAA-894 (**C**) and ATCC 29544 (**D**) in powdered infant formula.

To verify the potential of Chage1 as a biological control agent against *C. sakazakii* in food matrices, two strains (ATCC 29544 and BAA-894) were inoculated into powdered infant formula and milk samples containing Chage1 at an MOI of 10^5^. Bacterial survival was monitored for 8 h. At the optimal growth temperature of 37°C, bacterial counts in the control groups increased significantly over time. In contrast, Chage1 treatment resulted in a significant reduction in viable cell numbers in both matrices. Chage1 treatment significantly reduced bacterial counts from 2 to 8 h in milk and at 4 and 6 h in PIF (Fig. 4A and 4C). Moreover, *C. sakazakii* ATCC 29544 exhibited a rapid and pronounced decrease, showing highly significant reductions as early as 2 h, with mean of 2.86 ± 0.80 and 4.78 ± 1.25 log CFU/ml within 2 h in milk and PIF, respectively (Fig. 4B and 4D).

### Genomic Analysis

The complete genome of phage Chage1 comprised 52,653 bp of double-stranded DNA, with a 45.74% average G + C content and 72 putative ORF. Functional ORFs were categorized into six groups according to their function: phage DNA packaging, phage structure, host lysis, nucleotide metabolism, additional functions, and hypothetical proteins (Fig. S3). As no lysogeny-related genes, such as integrases, recombinases, or repressors, were found, this suggests that Chage1 is a virulent phage. Additionally, no antibiotic resistance or virulence factor genes were detected in the Chage1 genome, which is a favorable characteristic in terms of food safety. Therefore, bacteriophage Chage1 is a promising biological control agent that can be employed to kill *C. sakazakii* strains. Interestingly, BLASTn analysis revealed that the genome of Chage1 was more similar to *Escherichia* and *Salmonella* phages, than to *Cronobacter* phages. The Chage1 genome shares 75% and 73% query cover, with DNA sequence identity of 83.59% and 76.60%, to *Escherichia* phage vB_EcoM_fHy-Eco03 (GenBank Accession No. MW602648) and *Salmonella* phage vB_SEnST11_KE26 (GenBank Accession No. PP856725), respectively. In addition, an average nucleotide identity (ANI) comparison revealed that Chage1 shares approximately 70% identity with other related phages, which is notably lower than the 95% threshold generally used to define the same species. This relatively low ANI value further supports the uniqueness of Chage1 at the genomic level.

**Fig. 5:**
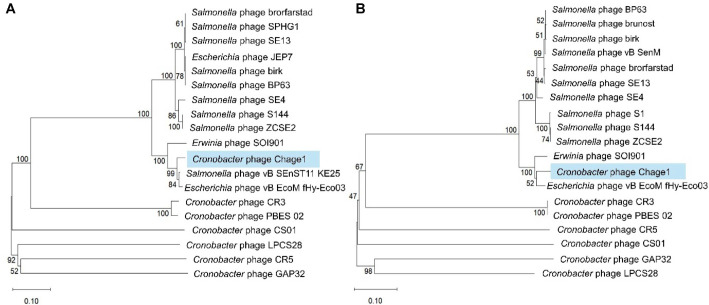
Comparative phylogenetic analysis. Phylogenetic trees were constructed based on the amino acid sequences of TerL (**A**) and MCP (**B**) from Chage1 and genetically related phages. The scale bars indicate the number of nucleotide substitutions per site, and the numbers at the nodes represent bootstrap probabilities.

To analyze the evolutionary relationship of Chage1, a phylogenetic analysis was conducted based on the amino acid sequences of terminase large subunit (TerL) and major capsid protein (MCP) (Fig. 5A and 5B). For this analysis, phages with high genomic similarity to Chage1 were selected, along with seven additional *C. sakazakii*-infecting phages. The assay revealed that Chage1 was evolutionarily closer to *Escherichia* phage vB_EcoM_fHy-Eco03 and *Erwinia* phage SOI901 (GenBank Accession No. PP700703.1), whereas *Salmonella* phages with similar whole-genome sequences showed numerous branches with relatively low bootstrap values. This indicated uncertain evolutionary connections between these phages. Specifically, Chage1 exhibited substantial differences from other *Cronobacter* phages in terms of terminase large subunit and major capsid protein. This exhibited that Chage1 may represent a new phage lineage that is genetically distinct from the existing *Cronobacter* phages.

### Identification of Endolysin LysCG1

**Fig. 6:**
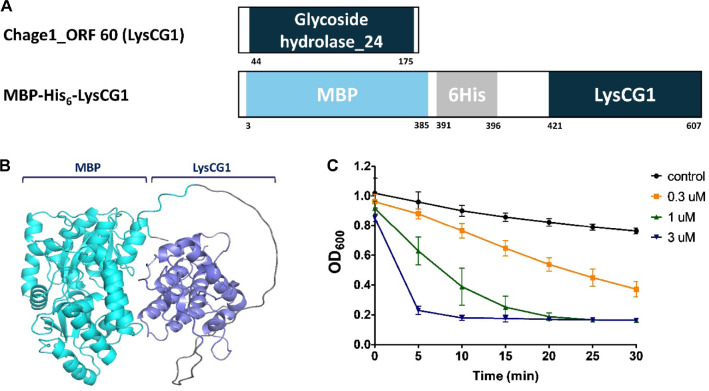
Characteristics of LysCG1. Modular structure (**A**) and tertiary structure of MBP-tagged LysCG1 (**B**) predicted by Alphafold2. (**C**) Turbidity reduction of EDTA-pretreated *C. sakazakii* ATCC 29544 cells following treatment with different concentrations of LysCG1. Cells pretreated with EDTA and lysis buffer alone served as the negative control.

ORF 60 of the Chage1 genome was identified as an endolysin named LysCG1, and its lytic activity was evaluated to determine its potential as an antimicrobial agent against *C. sakazakii*. According to the conserved domain database analysis, LysCG1 was identified as a glycoside hydrolase (Pfam IPR002196) that cleaves 1,4-beta bonds between N-acetylglucosamine and N-acetylmuramic acid in peptidoglycan (Fig. 6A). A recombinant endolysin with enhanced solubility was constructed by fusing maltose binding protein (MBP) to the N-terminus of LysCG1 (Fig. 6A and 6B), and it was purified using affinity chromatography with the His-tag and Ni^+^ resin (Fig. S4). The purified endolysin was added to EDTA-pretreated *C. sakazakii* ATCC 29544 suspensions at varying concentrations to assess its ability to lyse bacterial cells. LysCG1 (3 μM) demonstrated rapid lytic activity within 5 min, and lower concentrations of 1 μM and 0.3 μM LysCG1 exhibited excellent lytic ability within 30 min (Fig. 6C). In contrast to the very narrow host specificity of phage Chage1, LysCG1 displayed broad lytic activity against Gram-negative bacteria, including *C. sakazakii*, whereas it did not show lytic activity against Gram-positive bacteria (Table 1).

## Discussion

*Cronobacter sakazakii*, *Listeria monocytogenes*, and *Salmonella* spp. are among the top three pathogens currently associated with post-processing contamination of dairy products [22]. Phages are host-specific and do not infect non-targeted microorganisms in food, rendering them a promising tool for controlling this pathogen in dairy foods. In this study, a novel *C. sakazakii* phage (Chage1) was isolated and its antimicrobial efficacy in regulating *C. sakazakii* was assessed in PIF and milk. Furthermore, the antibacterial efficacy of its endolysin LysCG1 against a broad spectrum of *Cronobacter* spp. pathogens was evaluated.

Chage1 infected the 4 strains of *C. sakazakii* including BAA-894, which is typically considered less susceptible to phage infections. Additionally, Chage1 produced inhibition zones on several *Cronobacter* and *Salmonella* strains without forming visible single plaques. The observation indicates that phage activity was present but not accompanied by productive replication. Such inhibition without plaque formation has been reported previously [31, 32] and may arise from abortive infections, host defense systems, or enzymatic activities such as tailspike depolymerases that prevent bacterial proliferation in the absence of plaque development.

Thermal and pH tolerance are important properties of phages to consider for their application in food matrices. The titer of Chage1 did not decrease considerably after 3 h of treatment at 4–50°C; however, the titer decreased over time at 60°C and was completely inactivated after 2.5 h. Chage1 demonstrated superior pH stability across a wider range (pH 4–11) than that of other *Cronobacter*-infecting phages, such as PBES (pH 6–10) [33] and LPCS28 (pH 4–10) [22]. This suggests that Chage1, which is stable across a broad pH range, may have diverse applications in the food industry.

The antibacterial efficacy of Chage1 was evaluated in PIF inoculated with two distinct *C. sakazakii* strains at both 37°C and 4°C. At 37°C, Chage1 substantially reduced bacterial counts within 0–8 h post-infection, with the most notable effect observed against strain ATCC 29544, which exhibited a maximal 4-log reduction. However, at 4°C, no significant bacterial growth or reduction was observed throughout the experiment (Fig. S2). These findings are consistent with those reported for phage LPCS28, which also showed no bacterial regrowth or phage-induced killing at 4°C, while demonstrating a delayed bactericidal effect at 37°C beginning at 6 h post-infection [22]. In contrast, phage JK004 achieved complete eradication of *C. sakazakii* in PIF between 4–16 h at 37°C and also exhibited a moderate reduction (~1 log) at 4°C [16]. Phages CR5 and A24 both completely eliminated *C. sakazakii* at 37°C within the same timeframe; however, CR5 was not tested at 4°C, whereas A24 and CBT2 showed no detectable activity at that temperature [2, 17, 34]. Compared to these previously reported phages, Chage1 demonstrated a rapid onset of lytic activity at 37°C but limited activity at 4°C, a pattern similar to that of LPCS28 [22] and A24. This temperature dependency suggests that Chage1 may be particularly suitable for application during preparation or under feeding conditions, rather than for long-term cold-chain biocontrol. Nevertheless, since PIF is generally processed and stored at temperature below 25°C, further studies should evaluate the antimicrobial efficacy of Chage1 under simulated storage and preparation conditions (20−40°C).

Genome sequencing analysis revealed that Chage1 does not contain any antibiotic resistance or toxin factor genes, which is a desirable characteristic that enhances the safety of using this phage in food production. Therefore, bacteriophage Chage1 is a promising biological control agent that can be employed to lyse *C. sakazakii* strains. Interestingly, comparative genomic analysis of Chage1 and seven other reported *C. sakazakii* phages revealed that Chage1 is clearly distinct from other *Cronobacter* phages. The S144 phage (GenBank accession No. MT663719.1), which co-infects both *Salmonella* species and *Cronobacter* [35], exhibited the greatest similarity (query 32%, identity 76%) with Chage1 among the *Cronobacter*-infecting phages. However, limited query coverage indicates substantial genomic divergence between the two phages. Moreover, the conserved regions were mainly restricted to core structural and replication genes, such as MCP, TerL, DNA polymerase, and tail assembly chaperones, which are not directly involved in host specificity (Fig. S5). In contrast, tail fiber proteins exhibited pronounced sequence divergence among Chage1 and related phages. Specifically, the tail fiber protein of the closely related *E. coli* phage fHy-Eco03 shared 78% sequence identity with that of Chage1, whereas no detectable homolog was identified in the *Salmonella* phage vB_SEnST11_KE26. In addition, the tail fiber protein of phage S144, which infects both *Cronobacter* and *Salmonella*, showed only 68% identity. These differences in tail fiber proteins may partially explain the distinct host ranges observed among these genetically related phages (Fig. S6).

Given its unique host range and effective lytic activity, Chage1 may serve as a valuable component in phage cocktails targeting *C. sakazakii*. Although *C. sakazakii* is a major pathogenic species, the *Cronobacter* genus also comprises other medically important species, including *Cronobacter malonaticus*, *C. turicensis*, *C. universalis*, *C. muytjensii*, and *C. dublinensis* [36]. To broaden the spectrum of activity against these diverse pathogens, phage-derived endolysins which often exhibit wider host ranges than those of their parental phages may offer a promising approach [37]. Therefore, this study sought to identify a Chage1-derived endolysin and assess its antimicrobial activity as a potential alternative or complementary antimicrobial agent to Chage1.

To use the Chage1 endolysin, the Chage1_ORF 60 sequence was inserted into pET28a and expressed in *E. coli*; however, the solubility of the protein was low. To improve solubility, the MBP sequence was inserted at the N-terminus and a higher concentration of protein was obtained. When purified LysCG1 was added alone to Gram-negative bacterial cells, turbidity was not reduced. This may be because the outer membrane of Gram-negative bacteria prevents endolysin from reaching the peptidoglycan layer, thereby precluding bacterial lysis. This is why many endolysins are ineffective against Gram-negative bacteria. Correspondingly, notable LysCG1 lytic activity was observed when bacterial cells were pretreated with the cell membrane permeabilizer EDTA. LysCG1 exhibited antimicrobial activity against *C. sakazakii* as well as *C. malonaticus*, *C. turicensis*, *C. universalis*, *C. muytjensii*, and a wide range of Gram-negative bacteria. However, chelating agents, such as EDTA, cannot be utilized in food applications. Therefore, to effectively apply LysCG1 in food, strategies such as combining it with organic acids or engineering fusion peptides that enhance outer membrane permeability should be considered [38].

## Conclusion

This study reports the novel lytic bacteriophage Chage1, which specifically targets *C. sakazakii* including the BAA-894 strain. Chage1 demonstrated robust stability within a temperature range of 4–50°C and under pH conditions spanning 4–11. At an MOI of 10^5^, Chage1 effectively inhibited *C. sakazakii* growth in PIF and milk samples for up to 8 h at 37°C. Whole-genome sequencing revealed that Chage1 possesses a 52,653 bp DNA genome encoding 72 predicted ORFs. The Chage1 genome lacks virulence factors or antibiotic resistance genes, thus suggesting its potential as an antimicrobial agent for food safety applications. Furthermore, the endolysin LysCG1 derived from Chage1 exhibited a broader lytic spectrum than that of Chage1 by effectively lysing diverse EDTA-pretreated Gram-negative bacterial cells. Owing to its considerable antibacterial efficacy in food matrices and its unique host range, Chage1 demonstrates promising potential as a biocontrol agent for managing *C. sakazakii*, thereby enhancing food safety.

## Supplemental Materials

Supplementary data for this paper are available on-line only at http://jmb.or.kr.



## Figures and Tables

**Table 1:** Host range of Chage1 and its endolysin.

Species	Strain	Chage1	LysCG1
*Cronobacter sakazakii*	ATCC 29544	+	+++
	ATCC 29004	-	++
	ES15	-	++
	BAA-894	+	++
	Isolate (Seoul PIF)	+	++
	Isolate (Mail PIF)	I	+++
	Isolate 2 (whole milk powder)	+	++
	Isolate 31-3 (sun-sik)	I	+
	Isolate 2 (shrimp powder)	I	++
	Isolate 2-1 (powdered vegetable)	-	++
	Isolate 18-2 (powdered vegetable)	-	++
*C. muytjensii*	ATCC 51329	I	+
*C. turicensis*	DSM 18703	I	+++
*C. malonaticus*	DSM 18702	I	++
*Salmonella enterica* serovar infantis		I	+
*S. enterica* Typhimurium	SL1344	-	++
*S. enterica* Typhimurium	LT2	-	+
*S. enterica* Enteritidis	ATCC 13076	-	+
*S. enterica* Typhi	Ty2-b	-	++
*S. enterica* Dublin	1B 2973	-	++
*S. salamae*	KCCM 41762	-	++
*S. indica*	KCCM 41759	I	+
*E. coli*	MG 1655	-	++
*E. coli* O157:H7	OE50	-	++
*Klebsiella pneumoniae*	KCTC 2242	-	++
*Yersinia enterocolitica*	ATCC 55075	-	+
*Shigella flexneri* 2a	Strain 245T	-	++
*Citrobacter freundii*	14766	-	+
*Bacillus cereus*	ATCC 11306	-	-
*Listeria monocytogenes*	ATCC 15313	-	-
*Staphylococcus aureus*	Newman	-	-
*Enterococcus faecalis*	ATCC 10100	-	-
*Bifidobacterium adolescentis*	ATCC 15703	-	-
*Lactobacillus paracasei*	ABI1	-	-
*Lactobacillus rhamnosus*	ATCC 53103	-	-

Phage host range was assessed as follows: +, plaque formation; -, no plaque formation; I, growth inhibition without plaque formation. Endolysin activity was categorized based on relative lytic activity: -, 0–10%; +, 11–40%; ++, 41–70%; +++, 71–100%.
