# Supplementary material for: Evaluation of the Novel Bacteriophage Chage1 and Its Endolysin LysCG1 as Biocontrol Agents against Cronobacter sakazakii in Foods
Source: J Microbiol Biotechnol. 2026 Mar 26;36:e2601065. doi: 10.4014/jmb.2601.01065 (PMC13036506; doi:10.4014/jmb.2601.01065)
Supplement: Supplementary file 1 [file jmb-36-e2601065-supple.pdf]

J. Microbiol. Biotechnol. 2026. 36: e2601065

https://doi.org/10.4014/jmb.2601.01065

Supplemental Figures

Evaluation of the novel bacteriophage Chage1 and its endolysin LysCG1 as biocontrol agents against *Cronobacter sakazakii* in foods

Jin Seo, and Minsuk Kong*

Department of Food Science and Biotechnology, Seoul National University of Science and Technology, Seoul 01811, Republic of Korea

*** Correspondence: kongmin1@seoultech.ac.kr**

Table of Content

1. Supplementary figures 2

Fig. S1 2

Fig. S2 3

Fig. S3 4

Fig. S4 5

Fig. S5 6

Fig. S6 7

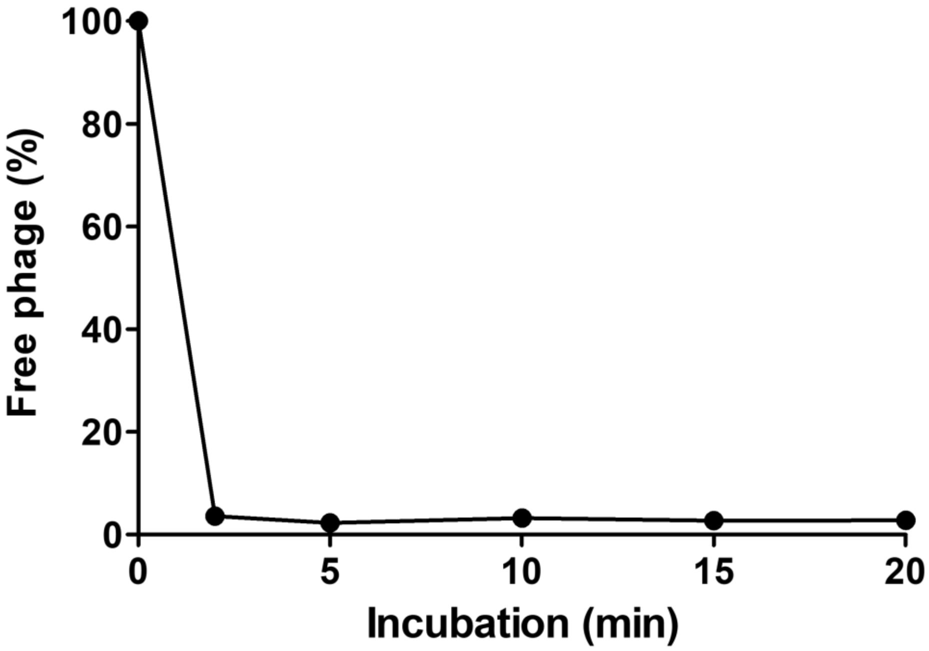

Fig. S1. Adsorption test of Chage1. it showed 90% of adsorption rate within only 2 min.

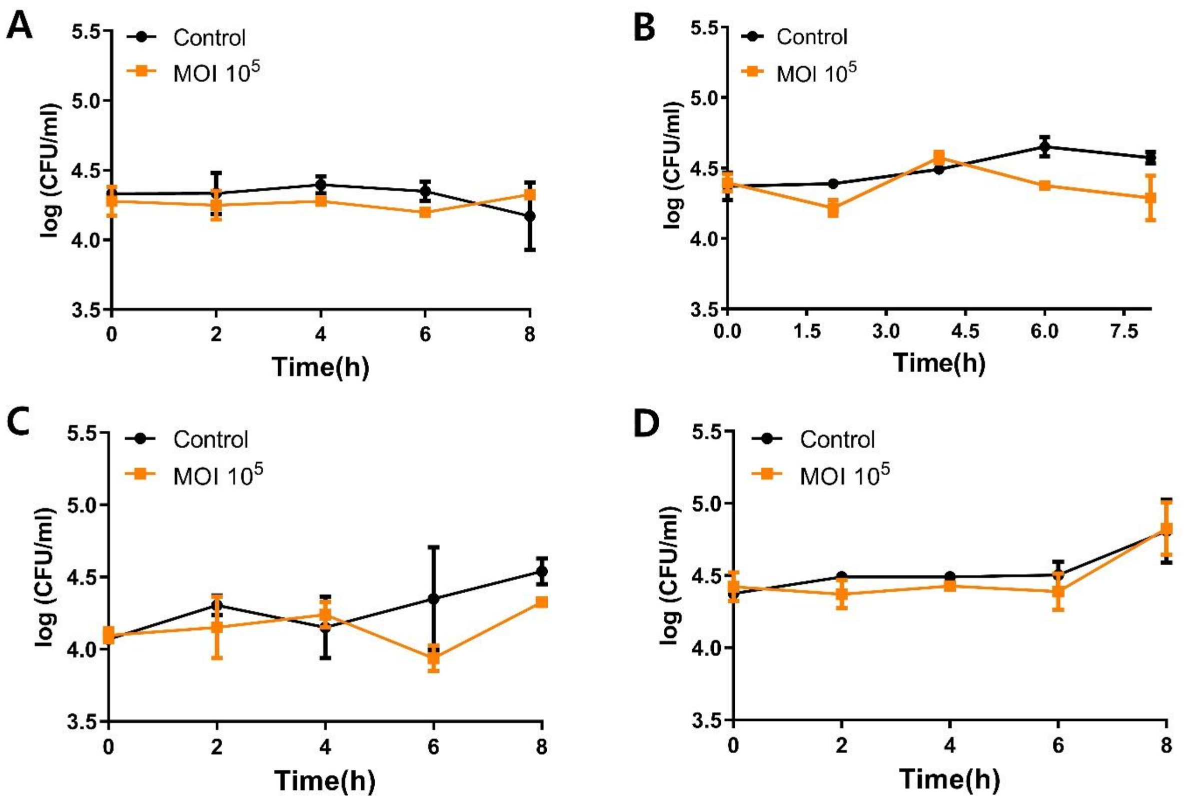

Fig. S2. Antimicrobial efficacy of Chage1 in food matrices at 4°C for 8 h. Chage1 demonstrated no detectable antibacterial activity against *C. sakazakii* BAA-894 **(A, C)** and ATCC 29544 **(B, D)** in sterilized skim milk **(A, B)** and in powdered infant formula **(C, D)**.

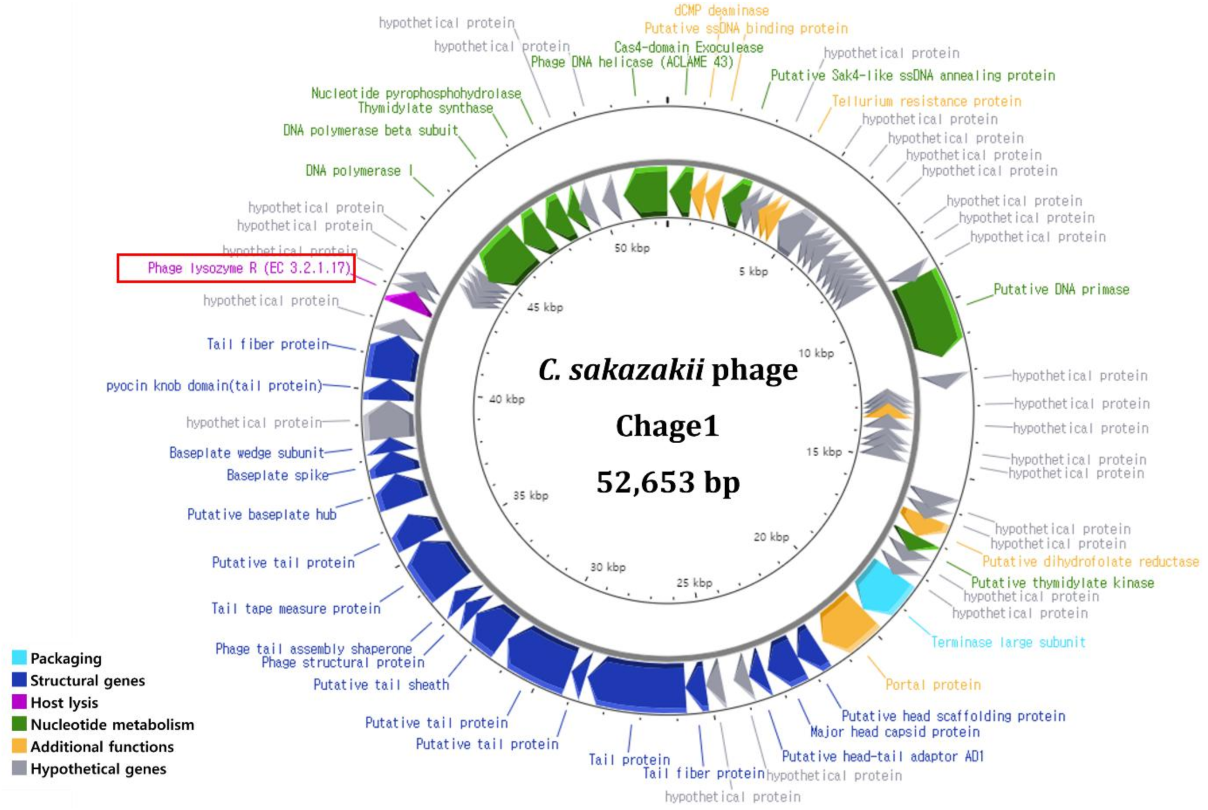

Fig. S3. Each color of ORFs indicates its function: sky blue, packaging; blue, structural genes; purple, host lysis; green, nucleotide metabolism; yellow, additional functions; and grey, hypothetical genes.

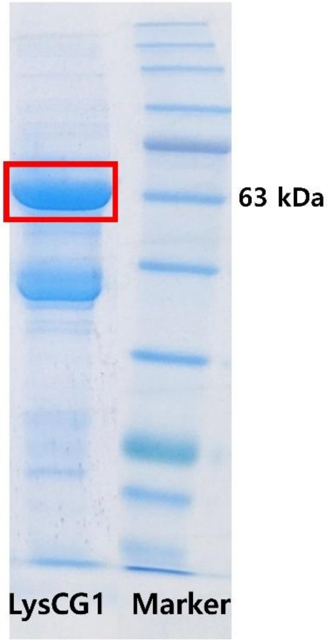

Fig. S4. SDS-PAGE gel image of LysCG1.

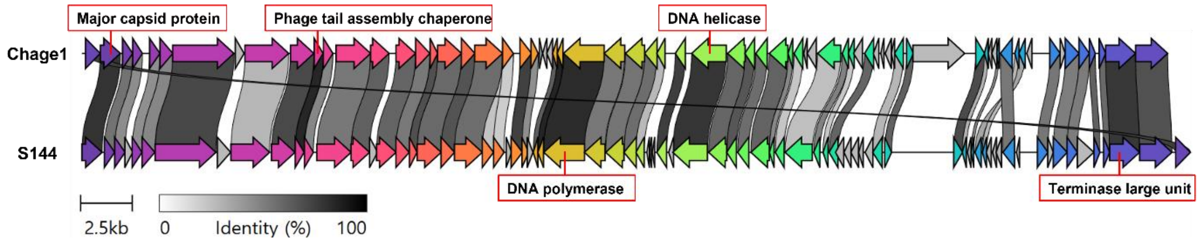

Fig. S5. Comparison between phage Chage1 and S144 genome.

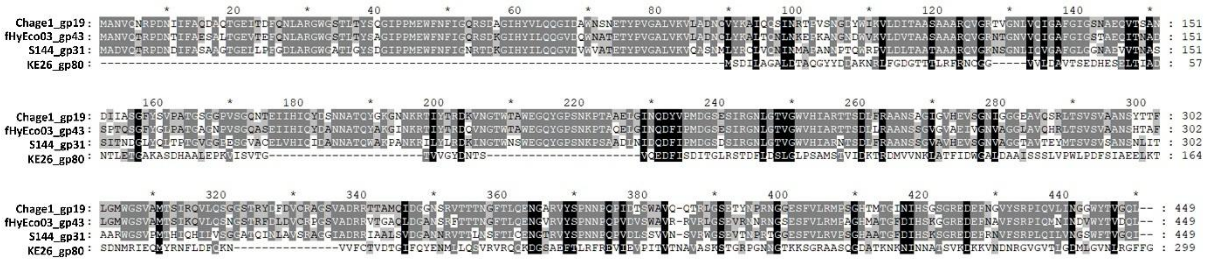

Fig. S6. Sequence alignment of tail fiber proteins from Chage1 and closely related phages. Amino acid sequences were aligned using ClustalX 2.0, and conserved residues are indicated by shaded regions.
